# Rare parasitic copepods (Siphonostomatoida: Lernanthropidae) from Egyptian Red Sea fishes

**DOI:** 10.1007/s11230-016-9665-5

**Published:** 2016-09-14

**Authors:** Hoda Hassan El-Rashidy, Geoffrey Allan Boxshall

**Affiliations:** 1 Department of Oceanography, Faculty of Science, Alexandria University, Moharram Bey, Alexandria, Egypt; 2 Department of Life Sciences, Natural History Museum, Cromwell Road, London, SW7 5BD UK

## Abstract

Two rare species of parasitic copepods belonging to the genus *Lernanthropus* de Blainville, 1822 (Siphonostomatoida: Lernanthropidae) are redescribed in detail, based on material collected from Red Sea fishes, caught at El-Tor, near Sharm El-Sheikh on the Red Sea coast of Egypt. Adult females of *Lernanthropus sanguineus* Song & Chen, 1976 were found on the gills of snapper *Lutjanus fulviflamma* (Forsskål). This species was known only from its original description based on material from Chinese waters. Adult females of *Lernanthropus triangularis* Pillai, 1963 were obtained from the gills of mojarra *Gerres oyena* (Forsskål). Both parasite species are new records for Egyptian Red Sea waters and both host records are new.

## Introduction

The family Lernanthropidae Kabata, 1979 currently comprises about 150 species belonging to eight genera (Boxshall & Halsey, 2004). The genus *Lernanthropus* de Blainville, 1822 is the largest of the family, containing approximately 120 valid species, all of which parasitise marine fish hosts belonging to at least 31 different teleost families (Boxshall & Halsey, 2004). Two species are reported in this account; the first, *Lernanthropus sanguineus* Song & Chen, 1976, was originally described from material collected from *Lutjanus sanguineus* (Cuvier) (family Lutjanidae) caught off the coast of China (Song & Chen, 1976). It is recorded here on a second lutjanid host, *L. fulviflamma* (Forsskål). The second species, *L. triangularis* Pillai, 1963, was originally described from material obtained from the gills of *Gerres filamentosus* Cuvier collected at Trivandrum, India (Pillai, 1963). In the present study *L. triangularis* was collected from the gill filaments of a second species of gerreid, *Gerres oyena* (Forsskål). The adult females of both of these rare species are redescribed in detail.

## Materials and methods

Host fish were purchased from local markets and examined for the presence of parasitic copepods. Copepods were removed from the host and preserved in 70% ethanol. The copepods were dissected and mounted in lactophenol as temporary slide preparations and examined on an Olympus microscope. Measurements were made using an ocular micrometer and drawings were made with the aid of a drawing tube. Morphological terminology follows Huys and Boxshall (1991). Host names were validated against FishBase (Froese & Pauly, 2016).

**Family Lernanthropidae Kabata, 1979**

**Genus*Lernanthropus*de Blainville, 1822**

***Lernanthropus sanguineus*Song & Chen, 1976**

*Host*: *Lutjanus fulviflamma* (Forsskål).

*Locality*: El-Tor (Red Sea), Egypt.

*Site on host*: Gill filaments.

*Material examined*: Three females stored in the collection of the first author.

*Prevalence*: 38% (3 infected hosts out of 8 examined).

### Description (Figs. 1–2)

**Fig. 1:**
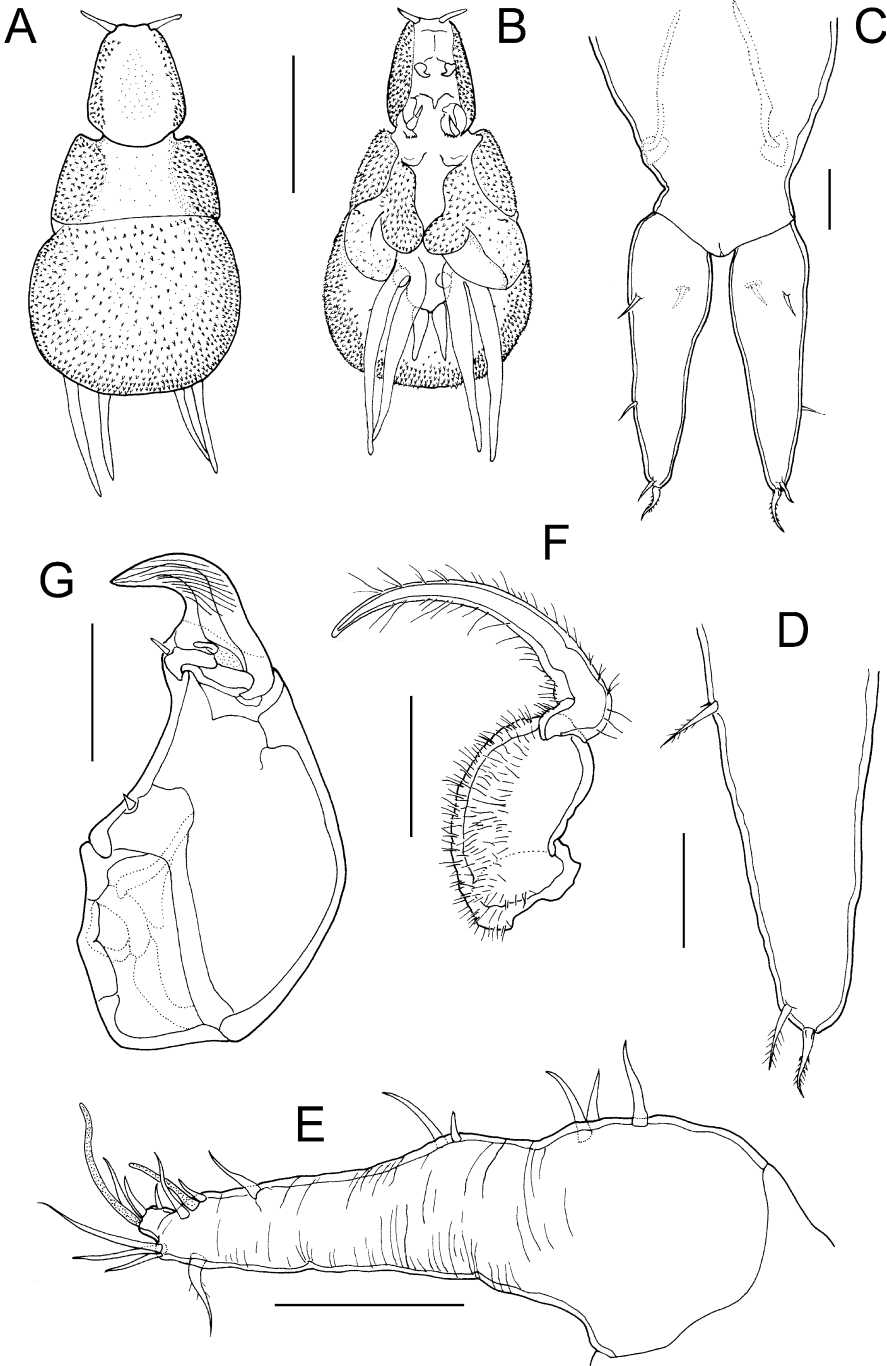
*Lernanthropus sanguineus* Song & Chen, 1976, female. A, Habitus, dorsal; B, Habitus, ventral; C, Genito-abdomen and caudal rami; D, Distal part of caudal ramus showing lateral and apical setae; E, Antennule; F, Parabasal flagellum; G, Antenna. *Scale-bars*: A, B, 1.0 mm; C, G, 100 µm; D–F, 50 µm

**Fig. 2:**
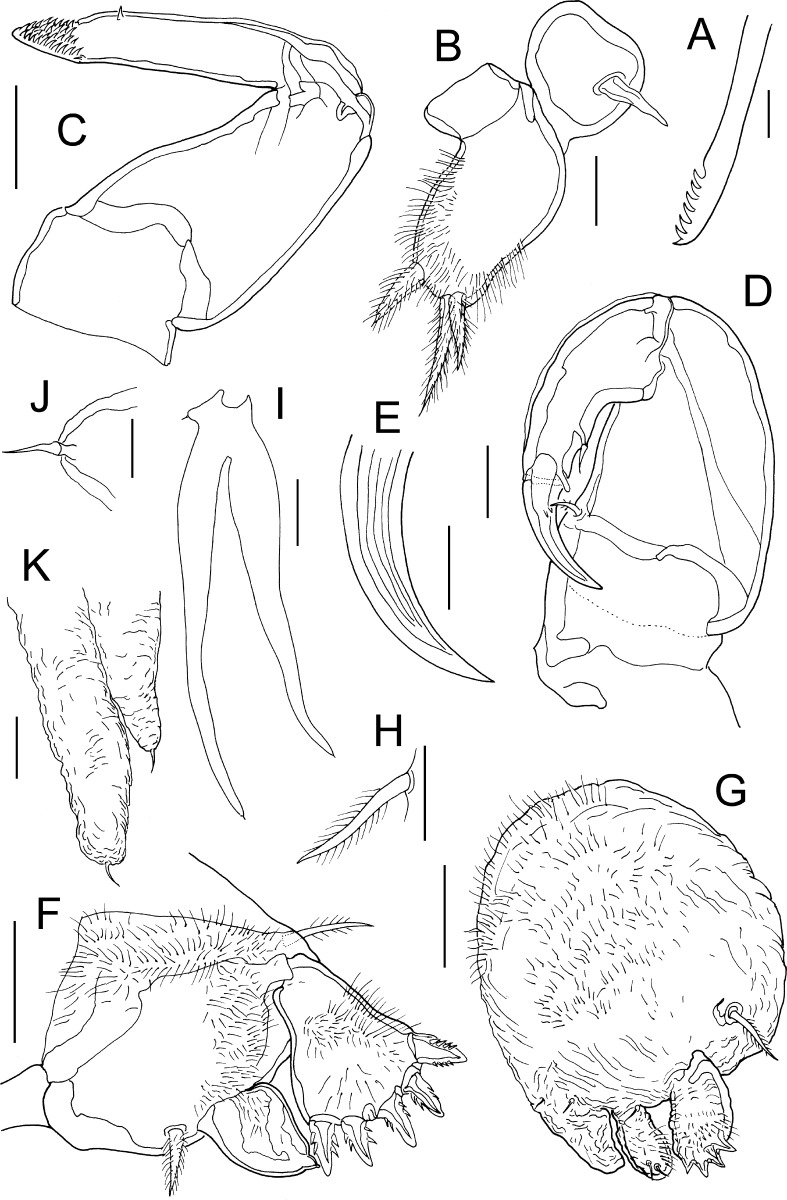
*Lernanthropus sanguineus* Song & Chen, 1976, female. A, Tip of mandible; B, Maxillule; C, Maxilla; D, Maxilliped; E, Terminal claw of maxilliped; F, Leg 1; G, Leg 2; H, Basal seta of leg 3; I, Leg 4; J, Basal seta of leg 4; K, Distal tips of lobate rami of leg 4. *Scale-bars*: A, B, E, H–K, 25 µm; C, D, F, G, 50 µm

*Adult female*. Body comprising cephalothorax and trunk (Fig. 1A, B). Mean body length measured from frontal margin of cephalothorax to posterior margin of dorsal plate 3.18 mm (based on 3 specimens). Cephalothorax *c*.1.2 times longer than wide; lateral margins produced ventrally into flanges on either side of cephalothorax (Fig. 1B). Lateral surfaces of cephalothorax densely ornamented with stout spinules. Trunk in dorsal view comprising subrectangular pedigerous somites anteriorly, and subcircular dorsal plate posteriorly; together *c*.1.3 times longer than maximum width. Anterolateral corners of trunk slightly produced into “shoulders” (Fig. 1A). Lateral surfaces of pedigerous somites and entire dorsal surface and ventral margins of dorsal plate densely ornamented with stout spinules. Dorsal plate with regularly convex posterior margin concealing entire posterior part of trunk, abdomen and caudal rami from dorsal aspect (Fig. 1A) and overlapping proximal parts of rami of leg 4. Genital complex (Fig. 1C) longer than wide and fused to short, broad abdomen. Pair of elongate caudal rami located posteriorly on abdomen; each ramus broadest at base and tapering distally; about 3.3 times longer than wide (Fig. 1D); armed with 5 small setae, 1 on dorsal and 1 on ventral surface proximally, 1 on lateral margin just distal to mid-length, and 2 at apex.

Antennule (Fig. 1E) unsegmented, proximal part bearing 5 setae on anterior surface, distal part armed with total of 10 setae plus 2 aesthetascs. Parabasal flagellum bipartite (Fig. 1F), with broad basal part and curved distal part, both parts ornamented with dense surface covering of long setules. Antenna (Fig. 1G) robust, comprising massive corpus bearing stout spiniform element on medial surface, and distal subchela with striated terminal claw bearing small process and small blunt element at base. Mandible with 7 marginal teeth on terminal blade (Fig. 2A). Maxillule (Fig. 2B) bilobate, smaller outer lobe (palp) tipped with spiniform element; larger inner lobe (praecoxa) ornamented with patches of long setules on surface and bearing 3 unequal hirsute elements apically. Maxilla (Fig. 2C) 2-segmented, comprising unarmed proximal syncoxa (lacertus) and distal basis (brachium); basis armed with single spiniform process subdistally and short apical claw ornamented with sharp denticles on inner surface. Maxilliped (Fig. 2D) 2-segmented, comprising massive corpus armed with seta on myxal surface, and distal subchela; subchela incorporating endopodal segments armed with spiniform element and blunt process on inner margin and curved terminal claw; surface of claw ornamented with linear striations (Fig. 2E).

Leg 1 biramous (Fig. 2F); protopod armed with slender outer pinnate seta and hiruste, spiniform inner seta; surface ornamented with patches of long setules; exopod 1-segmented, ornamented with patches of setules and armed with 5 stout spinulose spines along distal margin; endopod 1-segmented, tapering distally, bearing short stout apical process; surface ornamented with setules proximally. Leg 2 (Fig. 2G) with inflated protopod expanded into inner distal lobe adjacent to endopod; surface of protopod ornamented with long setules and 3 sensillae at base of inner lobe and endopod; armed with pinnate outer seta: both rami 1-segmented; exopod armed with 5 spines, a row of 4 stout spines along distal margin plus one small spine located anterior to distal row; endopod unarmed, ornamented with surface setules and 2 distal sensillae. Leg 3 forming large fleshy lamella extending ventrally (Fig. 1B), armed with pinnate outer basal seta (Fig. 2H); surface of inner lobe of lamella densely ornamented with stout spinules. Leg 4 (Figs 1B, 2I) biramous with both rami forming elongate, fleshy lobes; protopodal part armed with basal seta (Fig. 2J); outer lobe (exopod) slightly longer than inner (endopod), both lobes tipped distally with 1 setal vestige (Fig. 2K). Leg 5 absent.

### Remarks

The gross morphology of the females from the Red Sea is very similar to that described for *Lernanthropus sanguineus* by Song & Chen (1976), in the shape and relative proportions of the cephalothorax, trunk, and subcircular dorsal plate. The body lengths are also similar, with a mean length of 3.18 mm for the Red Sea females compared to a range of 2.85 to 3.08 mm given for female *L. sanguineus* (see Song & Chen, 1976). The caudal rami are elongate and taper distally from a wide base. The form of the bilobate leg 4, with its long slender lobes, is the same, and the distal parts of these lobes extend well beyond the posterior margin of the dorsal plate in both Red Sea and Chinese females. Despite these similarities, there are numerous differences in the detailed setation patterns of the limbs, for example, the caudal rami are shown as unarmed in Chinese females (cf. Song & Chen, 1976, Figure 2D) but carry 5 setae in Red Sea females, there are only 3 anterior setae on the proximal part of the antennule in the former compared to 5 in the latter. Numerous other differences in setation patterns are apparent for the antenna, maxilla, and maxilliped (cf. Song & Chen, 1976, figures 2f, 2h and 2i, respectively), and in all cases the differences can be summarised as the lack of setal elements in the illustrations of the Chinese specimens compared to the Red Sea females. Other obvious differences include the generally high level of surface spinulation found in the Red Sea females, for example on the outer lobe of the maxillule and on the protopods of legs 1 and 2, which was not figured for *L. sanguineus* (see Song & Chen, 1976). We consider that all of these setation and ornamentation differences can be attributed to observational factors since in 1976 less attention was paid to such details that we now consider to provide vital taxonomic and phylogenetic information. The remaining difficulty is the presence of the relatively large parabasal process (Fig. 1E) in the Red Sea females. Such a large process is more difficult to overlook. However, we identify the Red Sea material as *L. sanguineus* on the basis of the similarities in body morphology and basic limb construction. This is the first report of *L. sanguineus* from Egyptian waters and the first record since the original description forty years ago.

Apart from *L. sanguineus*, nine other species of *Lernanthropus* have been reported from lutjanid hosts: *L. bifidus* Pearse, 1951 from *Lutjanus analis* (Cuvier & Valenciennes) (see Pearse, 1951); *L. brevicephalus* Rangnekar, 1957 from *Lutjanus* sp., *Lutjanus malabaricus* (Bloch & Schneider) and *Lutjanus sanguineus* (see Rangnekar, 1957; Pillai, 1985; Ho et al., 2011); *L. eddiwarneri* Delamare Deboutteville & Nunes-Ruivo, 1954 on *Lutjanus fulgens* (Valenciennes) and *Lutjanus goreensis* (Valenciennes) (see Delamare Deboutteville & Nunes-Ruivo, 1954); *L. frondeus* C.B. Wilson, 1913 from *Lutjanus campechanus* (Poey) (as *Neomaenis aya*) (see Wilson, 1913); *L. kroyeri* van Beneden, 1851 from *Lutjanus griseus* (Linnaeus) (see Bere, 1936); *L. lativentris* Heller, 1865 from *Lutjanus vitta* (Heller, 1865 as *Mesoprion phaiotaeniatus*, see Kabata, 2005); *L. monodi* Delamare Deboutteville & Nunes-Ruivo, 1954 on *Lutjanus agenes* Bleeker (see Delamare Deboutteville & Nunes-Ruivo, 1954); *L. obscurus* C.B. Wilson, 1913 on *Ocyurus chrysurus* (Bloch) (see Wilson, 1913); *L. rathbuni* C.B. Wilson, 1922 on *Lutjanus griseus* (see Fuentes-Zambrano et al., 2003) and *Lutjanus campechanus* (as *Lutjanus blackfordii*) (see Causey, 1955); and *L. spiculatus* C.B. Wilson, 1913 on *Lutjanus synagris* (Linnaeus) (as *Neomaenis synagris*) (see Wilson, 1913; Bere, 1936; Pearse, 1951; Lagarde, 1991). The record of *L. kroyeri* van Beneden, 1851 from *Lutjanus griseus* (Linnaeus) in the Gulf of Mexico (see Bere, 1936) is unusual (see Kabata, 1979). It seems likely to us that this record is based on a misidentification of *L. rathbuni*, which is known from this host in Caribbean waters (Fuentes-Zambrano et al., 2003) and is very similar to *L. kroyeri* in the gross morphology of the female.

Only two other lutjanid-inhabiting species, *L. brevicephalus* and *L. lativentris*, are known from the Indo-Pacific. The former has been reported from three lutjanids including *Lutjanus sanguineus*, the type-host of *Lernanthropus sanguineus* (see Rangnekar, 1957; Pillai, 1985; Ho et al., 2011). These two copepods can be readily distinguished by the form of the third legs of the female which form flat elytra-like ventral plates in *L. brevicephalus* compared to the large fleshy bilobate lamellae which extend laterally and ventrally (Fig. 1B) in *L. sanguineus*, and by the 6-segmented antennules (compared with unsegmented antennules in *L. sanguineus*).

***Lernanthropus triangularis*Pillai, 1963**

*Host*: *Gerres oyena* (Forsskål).

*Locality*: El-Tor (Red Sea), Egypt.

*Site on host*: Gill filaments.

*Material examined*: One female stored in the collection of the first author.

### Description (Figs. 3–4)

**Fig. 3:**
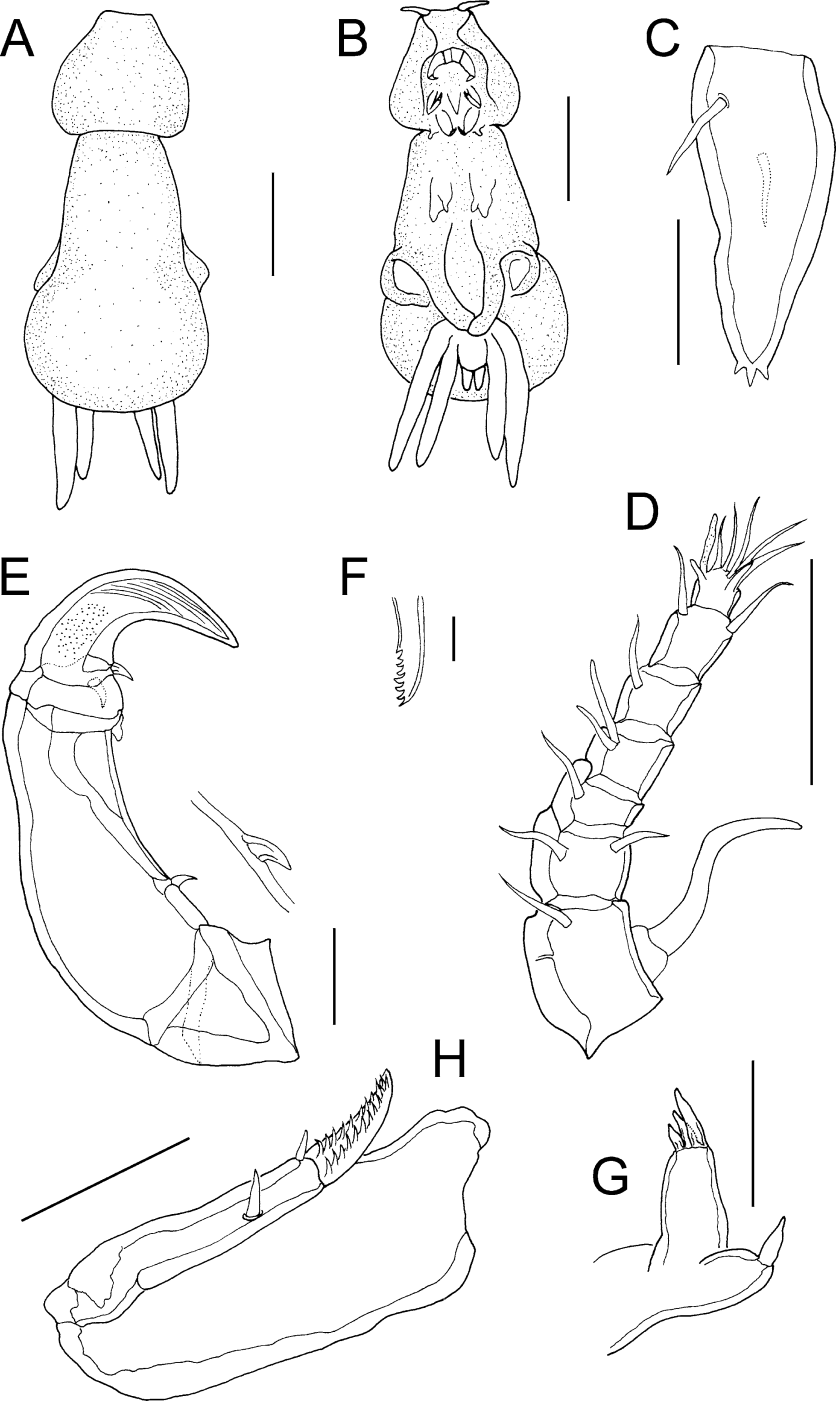
*Lernanthropus triangularis* Pillai, 1963, female. A, Habitus, dorsal; B, Habitus, ventral; C, Caudal ramus; D, Antennule and parabasal flagellum; E, Antenna; F, Tip of mandible; G, Maxillule; H, Maxilla. *Scale-bars*: A, B, 0.5 mm; E, 100 µm; C, D, G, H, 50 µm; F, 10 µm

**Fig. 4:**
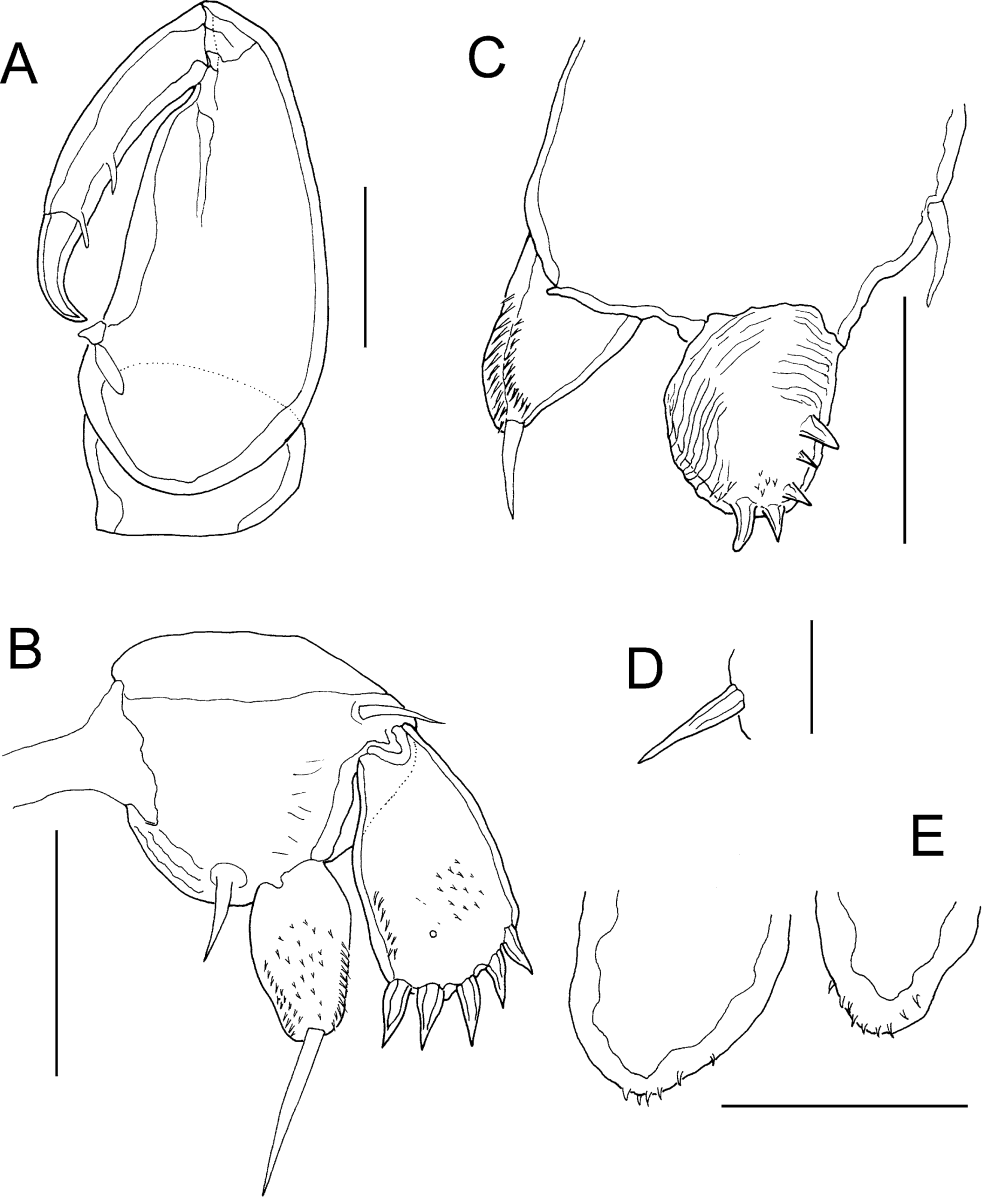
*Lernanthropus triangularis* Pillai, 1963, female. A, Maxilliped; B, Leg 1; C, Leg 2; D, Basal seta of leg 3; E, Distal tips of lobate rami of leg 4. *Scale-bars*: A–C, E, 50 µm; D, 25 µm

*Adult female*. Body comprising cephalothorax and trunk (Fig. 3A, B). Body length 2.1 mm, measured from frontal margin of cephalothorax to posterior margin of dorsal plate. Cephalothorax with lateral margins strongly rounded, narrowing anteriorly; produced ventrally into flanges on either side of cephalothorax (Fig. 1B), posterolateral corners slightly protruded; posterior margin slightly concave, about 2.5 times wider than anterior margin. Trunk in dorsal view comprising narrow pedigerous somites and broadly subcircular dorsal plate (Fig. 3A); together *c*.1.4 times longer than maximum width. Dorsal plate with regularly convex posterior margin concealing entire posterior part of trunk, abdomen and caudal rami from dorsal aspect (Fig. 3A) and overlapping proximal parts of rami of leg 4 (Fig. 3B). Abdomen small, not clearly differentiated from genital complex, bearing paired caudal rami posteriorly; each ramus tapering distally; about 2.3 times longer than wide (Fig. 3C); armed with 5 setae, 1 on dorsal and 1 on ventral surface proximally, and 3 short elements at apex.

Antennule (Fig. 3D) 7-segmented, setal formula 1, 2, 1, 1 + aesthetasc, 1, 2, 7 + aesthetasc. Parabasal flagellum originating adjacent to base of antennule; comprising long sigmoid distal process carried on swollen base (Fig. 3D). Antenna (Fig. 3E) slender, comprising small unarmed coxa; slender corpus (basis) bearing curved spiniform element proximally on medial margin; distal subchela with terminal claw and armed with small process plus 2 small setae proximally, surface of claw ornamented with minute pits and striations (Fig. 3E). Mandible armed with 7 marginal teeth on terminal blade (Fig. 3F). Maxillule (Fig. 3G) bilobate, smaller outer lobe (palp) tipped with 1 element; larger inner lobe (praecoxa) tipped with 3 unequal spiniform elements. Maxilla (Fig. 3H) 2-segmented, comprising robust unarmed syncoxa (lacertus) and distal basis (brachium); basis armed with large process plus smaller process at base of terminal claw; claw ornamented with sharp denticles on inner surface. Maxilliped (Fig. 4A) 2-segmented, comprising massive corpus armed with stout spiniform element on myxal margin, and distal subchela; subchela incorporating endopodal segments armed with 2 setae on concave inner margin, and bearing curved terminal claw (Fig. 4A).

Leg 1 biramous (Fig. 4B), protopod indistinctly divided into coxa and basis armed with outer and inner setae; exopod 1-segmented, broader distally, ornamented with patch of spinules on surface and armed with 5 robust spines along distal margin; endopod 1-segmented, pear-shaped, ornamented with patches of spinules on surface, and armed with long terminal seta nearly as long as ramus. Leg 2 (Fig. 4C) bearing outer seta on inflated protopod, both rami 1-segmented; exopod armed with 4 large terminal spines plus 1 smaller spine along outer and distal margins; endopod tapering distally, ornamented with patch of spinules along medial margin, armed with terminal seta about half length of ramus. Leg 3 forming large fleshy lamella extending ventrally (Fig. 3B); armed with dorsal outer basal seta (Fig. 4D). Leg 4 (Fig. 3B) biramous with both rami forming elongate fleshy lobes; outer lobe (exopod) slightly longer than inner (endopod), both lobes tipped distally with minute spinules (Fig. 4E). Leg 5 absent.

### Remarks

We identify this female as *L. triangularis* which was originally reported from *Gerres filamentosus* caught off Thiruvananthapuram (Trivandrum), Kerala state, India (Pillai, 1963). The female from the Red Sea is similar to the Indian material in shape and in the relative lengths of the cephalothorax, trunk, and subcircular dorsal plate, although the cephalothorax is slightly broader and has more strongly convex lateral margins in the Red Sea specimen. The body lengths are similar: 2.1 mm for the Red Sea female compared to 2.4 mm for the Indian material (Pillai, 1963). The caudal rami are elongate and taper both proximally and distally from their widest point near the base. The bilobate leg 4 has long slender lobes and the distal parts of these lobes extend well beyond the posterior margin of the dorsal plate in both Red Sea and Indian females. Additional similarities include the 7-segmented antennule, the form of the parabasal flagellum, the slender form of the antenna, and the setation patterns of the maxillule, maxilla and maxilliped.

The Red Sea female differs slightly from the Indian material of *L. triangularis* in the relative lengths of some of the setation elements of the rami of leg 2 and in the presence of 5 small apical spines on the exopod of the Red Sea female compared to only 4 in the Indian material (cf. Pillai, 1963, Figure 7I). These differences might be due to geographical variation, but equally might reflect the difficulties in observing some of these fine setation characters.

*Lernanthropus triangularis* has previously been reported only from *Gerres filamentosus*, so the host reported here, *G. oyena*, is a new host record. This is the first report of *L. triangularis* from Egyptian waters. No other lernanthropids have previously been reported from any species of *Gerres* Quoy & Gaimard, although the host of *L. cortezensis* Deets & Kabata, 1991 is the gerreid *Diapterus peruvianus* (Cuvier) (see Deets & Kabata, 1991). *Lernanthropus cortezensis* is a distinctive species characterised by the unique elongate laminiform third legs which are directed obliquely posteriorly and resemble the rami of the fourth legs (Deets & Kabata, 1991). It does not share a close similarity with *L. triangularis* either in gross body morphology or in characteristics of the limbs.
